# Variation in CYP2A6 Activity and Personalized Medicine

**DOI:** 10.3390/jpm7040018

**Published:** 2017-12-01

**Authors:** Julie-Anne Tanner, Rachel F. Tyndale

**Affiliations:** 1 Campbell Family Mental Health Research Institute, Centre for Addiction and Mental Health (CAMH), Toronto, ON M5T 1R8, Canada; julieanne.tanner@mail.utoronto.ca; 2 Department of Pharmacology and Toxicology, University of Toronto, Toronto, ON M5S 1A8, Canada; 3 Department of Psychiatry, University of Toronto, Toronto, ON M5T 1R8, Canada

**Keywords:** pharmacogenetics, pharmacogenomics, drug metabolism, CYP2A6, smoking, nicotine, genetic variation, SNP, inhibitor, inducer

## Abstract

The cytochrome P450 2A6 (CYP2A6) enzyme metabolizes several clinically relevant substrates, including nicotine—the primary psychoactive component in cigarette smoke. The gene that encodes the CYP2A6 enzyme is highly polymorphic, resulting in extensive interindividual variation in CYP2A6 enzyme activity and the rate of metabolism of nicotine and other CYP2A6 substrates including cotinine, tegafur, letrozole, efavirenz, valproic acid, pilocarpine, artemisinin, artesunate, SM-12502, caffeine, and tyrosol. CYP2A6 expression and activity are also impacted by non-genetic factors, including induction or inhibition by pharmacological, endogenous, and dietary substances, as well as age-related changes, or interactions with other hepatic enzymes, co-enzymes, and co-factors. As variation in CYP2A6 activity is associated with smoking behavior, smoking cessation, tobacco-related lung cancer risk, and with altered metabolism and resulting clinical responses for several therapeutics, CYP2A6 expression and enzyme activity is an important clinical consideration. This review will discuss sources of variation in CYP2A6 enzyme activity, with a focus on the impact of *CYP2A6* genetic variation on metabolism of the CYP2A6 substrates.

## 1. *CYP2A6* as a Pharmacogene

The human cytochrome P450 2A6 (CYP2A6) enzyme, primarily expressed in the liver, metabolizes several clinically relevant substrates [1,2]. However, there is extensive interindividual variation in CYP2A6 enzyme activity and thus the rate of metabolism of CYP2A6 substrates. The primary predictor of variable CYP2A6 activity is variation at the *CYP2A6* gene locus [3]. The gene that encodes the CYP2A6 enzyme is highly polymorphic, with more than 40 variants characterized to date (summarized at Pharmacogene Variation Consortium (PharmVar) at www.PharmVar.org. Other factors that contribute to variation in CYP2A6 expression and function include induction or inhibition by pharmacological, endogenous, and dietary substances, age-related changes, or interactions with other hepatic enzymes, co-enzymes, and co-factors [4,5,6,7,8,9]. 

Variation in CYP2A6 activity is an important clinical consideration as this enzyme is involved in the metabolism or bioactivation of clinical therapeutics, carcinogens, and dietary substances, including nicotine, tegafur, letrozole, efavirenz, *N*-nitrosonornicotine (NNN), 4-(methylnitrosamino)-1-(3-pyridyl)-1-butanone (NNK), and caffeine, among others [10,11,12,13,14,15,16]. Nicotine, the principle psychoactive component in cigarette smoke, is primarily metabolized by hepatic CYP2A6 (70–80%) [12], and nicotine’s major metabolite, cotinine (COT), is metabolized exclusively by CYP2A6 to *trans*-3′-hydroxycotinine (3HC) [17]. Due to the long half-life of COT and the 3HC formation dependency, the ratio of nicotine’s metabolites 3HC/COT (referred to as the nicotine metabolite ratio, NMR), is stable in current smokers and functions as a surrogate measure of the rate of nicotine metabolism and an in vivo biomarker of CYP2A6 enzyme activity [18]. The measurement of the NMR (described here as 3HC/COT) is the most common and validated approach for determining in vivo CYP2A6 enzyme activity among regular smokers, and can be measured in different biological matrices, including blood, saliva, and urine. The NMR, and its utility, as well as alternative ratios used in the literature are described in more detail in a later section. 

This review will discuss sources of variation in CYP2A6 enzyme activity, with a focus on *CYP2A6* genetic variation. We will also highlight the clinical relevance of variable CYP2A6 enzyme activity, as CYP2A6 variation is associated with smoking behavior, smoking cessation, tobacco-related lung cancer risk, and with altered metabolism and resulting clinical responses for several therapeutics. 

## 2. Variation in CYP2A6 Enzyme Activity

### 2.1. CYP2A6 Genetic Variation

An in vivo measure of CYP2A6 activity, the NMR, is highly heritable; monozygotic versus dizygotic twin studies indicate that 60–80% of the variation in the NMR (i.e., CYP2A6 activity) can be attributed to genetic influences [3,19]. *CYP2A6* genetic variation is the primary contributor to variation in CYP2A6 enzyme activity; those homozygous for genetically null *CYP2A6* alleles (e.g., CYP2A6*4/*4 genotypes) produce no 3HC [3]. In Genome Wide Association Studies (GWASs) of the NMR, the vast majority and the most highly significant genome-wide significant signals occur at, or very near to, the *CYP2A6* gene locus [3,19,20]. Further, numerous studies have employed sequencing and genotyping of the *CYP2A6* gene, along with in vitro and in vivo assessments of CYP2A6 expression and activity, to characterize functionally significant *CYP2A6* genetic variants, across many different ethnic/racial populations. We will focus on the common (minor allele frequency (MAF) > 1%) genetic variants that influence CYP2A6 expression and/or enzyme activity (i.e., functionally significant); these variants are described below and summarized in Table 1.

The majority of the functionally significant *CYP2A6* genetic variants result in a decrease in CYP2A6 expression and/or activity; however, the CYP2A6*1X2A and CYP2A6*1X2B (gene duplications) and CYP2A6*1B alleles are the main exceptions (see Table 1). The *CYP2A6* gene duplications result from an unequal crossover of *CYP2A6* and the adjacent *CYP2A7* gene during recombination [21,22]. CYP2A6*1X2A and CYP2A6*1X2B, which have breakpoints in intron 8 and 5.2–5.6 kb downstream of *CYP2A6*, respectively, are associated with faster nicotine metabolism (greater CYP2A6 activity), compared to the wild-type CYP2A6*1A allele [21,22]. The CYP2A6*1B allele is characterized by a 58 bp gene conversion with *CYP2A7*, occurring in the *CYP2A6* 3′-untranslated region (3′-UTR) [23]. This variant is associated with greater in vivo nicotine metabolism [23], possibly resulting from increased CYP2A6 mRNA stability and thus higher protein levels, relative to the wild-type (CYP2A6*1A/*1A) *CYP2A6* [24]; however, no differences in CYP2A6 mRNA, protein, or activity were observed between CYP2A6*1A/*1A and CYP2A6*1A/*1B or CYP2A6*1B/*1B genotype groups in a human liver bank [25]. CYP2A6*1B is also in linkage disequilibrium with decrease and reduced function variants, adding to the complication in assessing its activity. 

*CYP2A6* genetic variants that have MAFs >1% in one or more populations, and are associated with lower CYP2A6 expression and/or activity, include *2, *4, *5, *7, *9, *10, *12, *17, *18, *20, *21, *23, *24, *25, *28, and *35 (See Table 1). The majority of these *CYP2A6* polymorphisms are non-synonymous single nucleotide polymorphisms (SNPs) present in exons, whereas the *4, *9, *12, and *20 variants are deletion, non-coding, or hybrid alleles. CYP2A6*4 is a gene deletion resulting from an unequal crossover with *CYP2A7*. Similar to the duplication variant, there are multiple forms of the *4 variant (A-H), resulting from different crossover points [26]. The resulting lack of CYP2A6 mRNA expression is associated with a complete loss of CYP2A6 activity in individuals homozygous for the CYP2A6*4 allele [26,27]. CYP2A6*9 is a SNP present in the TATA box of the *CYP2A6* promoter region, which also results in decreased CYP2A6 mRNA expression and subsequently lower enzyme activity, but to a lesser degree than CYP2A6*4 [28]. A crossover between the *CYP2A6* and *CYP2A7* genes produces the CYP2A6*12 hybrid allele, which is composed of exon 1 and 2 from *CYP2A7* and exons 3–9 from *CYP2A6*; CYP2A6*12 has 10-amino acid substitutions and decreased CYP2A6 activity [29]. The CYP2A6*20 allele possesses a two-nucleotide deletion in exon 4 that results in a frame shift, a truncated CYP2A6 protein, and substantially reduced CYP2A6 activity [30].

Of the nonsynonymous coding region SNPs, *5 (G479V), *7 (I471T), *18 (Y392F), *21 (K476R), *23 (R203C), *24 (V110L and N438Y), *25 (F118L), *28 (N418D and E419D), and *35 (N438Y) are associated with moderately decreased CYP2A6 enzyme activity [31,32,33,34,35,36], whereas *2 (L160H), *10 (I471T and R485L), and *17 (V365M) result in a substantial decrease or complete loss of CYP2A6 activity toward nicotine, similar to *4 and *20 [32,37,38,39]. The impact of these *CYP2A6* genetic variants on in vitro coumarin 7-hydroxylation, as well as nicotine-C oxidation (as summarized in Table 1), has additionally been characterized by Hosono et al., 2017 [40]; with the exception of CYP2A6*28, variants were associated with decreased nicotine C-oxidation, consistent with the direction of effect provided in Table 1. Of note, an earlier study demonstrated that individuals with the CYP2A6*1/*28 genotype have lower NMR (in vivo measure of nicotine metabolism) compared to CYP2A6*1/*1 genotype individuals [34], whereas Hosono et al. showed that there was no difference in nicotine C-oxidation (in vitro measure of nicotine metabolism) between the CYP2A6.1 and CYP2A6.28 constructs. 

Stemming from recent GWASs of the NMR (CYP2A6 activity phenotype) and improvements in sequencing technologies, novel *CYP2A6* genetic variants have been identified and characterized with respect to their impact on CYP2A6. The first GWAS of the NMR and meta-analysis of NMR GWASs in Finnish smokers identified three independent signals following conditional analyses on chromosome 19, the location of the *CYP2A6* gene, that were associated with the NMR: rs56113850, rs113288603, and esv2663194 (i.e., CYP2A6*12) [3]. Of note, the reduce-of-function CYP2A6*2 SNP was in LD with all three independent signals (D’ = 1 for all). The rs56113850 SNP was the top hit (*p* = 5.77 × 10^−86^, lowest *p*-value, most highly significant) associated with the NMR, and is located in intron 4 of *CYP2A6*. This SNP (C vs. reference T allele) is associated with higher CYP2A6 enzyme activity in this and other studies, including a multi-ethnic cohort NMR GWAS, a laboratory-based CYP2A6 phenotype GWAS [20,44], as well as in a human liver bank study and a clinical trial cohort [45]. The rs56113850 SNP accounted for approximately 14–22% of the variation in the NMR among Finnish smokers. The rs113288603 SNP was identified as an independent signal following conditional analyses with the rs56113850 SNP, and accounted for less than 1% of the variation in the NMR. The rs113288603 SNP is located 5′ of *CYP2A6* and was associated with lower NMR among Finnish smokers (T vs. reference C allele, *p* = 1.32 × 10^−9^, β = −0.47). The third top hit, esv2663194, corresponds to the previously established CYP2A6*12 hybrid of *CYP2A6* and *CYP2A7*, described above. This variant accounted for approximately 3–8% of the variation in the NMR among Finnish smokers, and it was associated with lower NMR (*p* = 3.34 × 10^−23^, β = −1.08), which replicates findings from previous studies of CYP2A6*12 [4,29].

In a GWAS of the NMR and a meta-analysis of NMR GWASs in African American smokers [46], three independent signals on chromosome 19 were identified that were associated with the NMR: rs12459249, rs111645190, and rs185430475. Of note, associations reported are for non-logged NMR. The top overall hit (lowest *p*-value), rs12459249, is located 3′ of the *CYP2A6* gene and was associated with higher NMR (C vs. reference T allele, *p* = 1.47 × 10^−39^, β = 0.59), accounting for more than 15% of the variation in the NMR. This SNP was still significantly associated with the NMR after controlling for the CYP2A6*17 variant, described above, that is common among African Americans (see Table 1). Of note, a meta-analysis of GWASs from a relatively small study using an alternative type of laboratory-based NMR in African, Asian, and European ancestry individuals also identified the rs12459249 SNP as a top hit associated with the NMR, as well as the rs56113850 SNP, as described above [20]. The second independent signal in African Americans identified by Chenoweth and colleagues was rs111645190, which is located 5′ of *CYP2A6* and was associated with lower NMR (A vs. reference G allele, *p* = 1.19 × 10^−11^, β = −0.42), accounting for approximately 3–5% of the variation in the NMR. The third independent signal, rs185430475, is a SNP located 3′ of *CYP2A6* that was associated with higher NMR (G vs. reference C allele, *p* = 1.94 × 10^−8^, β = 1.27), although not significantly in the meta-analysis. Unlike the rs12459249 SNP, rs111645190 and rs185430475 (second and third top hits) were not genome-wide significant after controlling for the CYP2A6*17 SNP, suggesting that these SNPs may be tagging the *17 variant and possibly do not exert an independent effect on CYP2A6 activity. It should be noted that findings from GWASs of one ethnic population may not be generalizable to other ethnic groups, as evidenced by the difference in hits between European ancestry and African ancestry GWASs, where approximately 60% of the hits in the African ancestry meta-analysis were not genome-wide significant among the Finnish cohort [3,46].

Using a *CYP2A6*-specific next-generation sequencing approach, we have identified seven novel variants that were significantly associated with variation in CYP2A6 expression and enzyme activity in vitro in a human liver bank [45]. One of the seven variants is the rs56113850 SNP that was identified as a top hit in multiple GWASs of the NMR, detailed above. The other six novel SNPs were: rs57837628, rs7260629, rs7259706, rs150298687 (merged into rs4803381), rs28399453, and rs8192733. All seven SNPs are located in non-coding regions of the *CYP2A6* gene, including 5′ of the gene, in introns, and in the 3′-UTR; all of the SNPs were associated with increased CYP2A6 enzyme activity. Six of the seven novel SNPs were in moderate-to-high linkage disequilibrium (LD) with each other, with r^2^ values ranging from 0.36 to 0.94 among White subjects in a human liver bank. Due to the high LD of two pairs of SNPs, a simplified five-SNP, as opposed to seven-SNP, diplotype was derived, which was associated with in vitro CYP2A6 activity in the liver bank and in vivo CYP2A6 enzyme activity (determined by the NMR) in a population of treatment-seeking smokers. 

Developing a CYP2A6 phenotype prediction method that considers both established and the recently identified novel *CYP2A6* genetic variants will be important in accounting for more of the variation in CYP2A6 enzyme activity, and patients’ responses to therapeutic compounds that are CYP2A6 substrates. 

### 2.2. Ethnic/Racial Differences in CYP2A6 Genetic Variation and CYP2A6 Activity

Different ethnic/racial groups exhibit distinct patterns of *CYP2A6* genetic variation, as summarized in Table 1. Some *CYP2A6* alleles are more common, or have thus far only been observed in specific ethnic/racial populations. For example, the CYP2A6*7 allele has been found predominantly in Asian populations [47], and, to date, CYP2A6*17, *20, *23-25, and *28 have been identified in people of African descent [35,48,49]. Despite differences in allele frequencies, the impact of each allele on CYP2A6 activity is similar across ethnic/racial populations. For example, a similar decrease in CYP2A6 enzyme activity of 33% and 39%, respectively, is observed in White and African American smokers with the CYP2A6*1/*9 genotype compared to CYP2A6*1/*1 (wild-type genotype) [50]. That said, it is still possible that alleles are associated with different phenotypes in different ethnic groups; for example, this could occur due to linkage with other genetic variants in one but not all populations.

Asian and African American populations tend to have higher frequencies of *CYP2A6* decrease- or loss-of-function genetic variants, and consequently exhibit lower overall CYP2A6 activity compared to White populations [47,48,51]. For example, compared to White individuals, Japanese American smokers have, on average, half the rate of CYP2A6 enzymatic activity (measured by urinary total 3HC/free COT) [50]. Similarly, after chewing nicotine gum, Japanese non-smokers exhibit nearly half the average CYP2A6 activity (measured by plasma cotinine/nicotine) compared to White non-smokers [45]. These data are consistent with the much higher allele frequencies of *CYP2A6* decrease- and loss-of-function genetic variants among the Japanese (overall frequency of these variant alleles in each population: 50% in Japanese, 12% in White individuals), and higher prevalence of individuals with decrease- and loss-of-function *CYP2A6* genotypes (overall frequency of individuals possessing decrease- and loss-of-function *CYP2A6* genotypes: 72% of Japanese, 21% of White individuals) [48]. These trends also extend to other Asian populations. Chinese American smokers exhibit significantly lower total and non-renal clearance of both nicotine and cotinine, indicative of lower CYP2A6 enzyme activity, compared to White smokers [52]. Likewise, CYP2A6 activity (salivary NMR) was significantly lower in Asian than in White adolescents (ages 13–17) [53]. 

Among African Americans, there are also more *CYP2A6* genotype reduced metabolizers compared to in White populations (overall frequency of individuals possessing reduce- and loss-of-function *CYP2A6* genotypes: 38% of African Americans, 21% of White individuals) [48]. Consistent with these higher frequencies, African Americans exhibit significantly lower non-renal clearance of cotinine compared to White individuals [54], suggestive of slower metabolism, mediated by CYP2A6. This is supported by a separate observation of lower overall CYP2A6 enzyme activity (measured by plasma NMR) among African American compared to White smokers [55]. Compared to White individuals, African American adolescent (ages 13–17) smokers also have significantly slower CYP2A6 activity (lower salivary NMR) [53].

Compared to White individuals, Alaska Natives also have higher frequencies of certain decrease- and loss-of-function *CYP2A6* genetic variants (higher CYP2A6*4, CYP2A6*9, and CYP2A6*10 allele frequencies), and these variants are similarly associated with lower CYP2A6 activity among Alaska Natives, as in other ethnic/racial groups [56]. The *CYP2A6* gene deletion (the CYP2A6*4 allele) is present at a frequency of nearly 15% in Yupik Alaska Native people, compared to approximately 1% in White individuals. Similarly, the frequency of the CYP2A6*10 haplotype was 2% in this population, but has not been reported to occur in White individuals. Despite the higher frequencies of these slower CYP2A6 activity alleles, Alaska Native smokers exhibit faster overall CYP2A6 activity (measured by plasma NMR) compared to White smokers. This difference is further increased when comparing CYP2A6 activity among Alaska Native and White subjects with the CYP2A6*1/*1 (wild-type) genotype (i.e., excluding subjects with known decrease- and loss-of-function *CYP2A6* genetic variants). The higher observed CYP2A6 activity (rate of nicotine metabolism) is not accounted for by known gain-of-function *CYP2A6* genotypes, and therefore other factors (novel genetic variation, dietary inducers, environmental exposures, etc.) may be contributing to the faster CYP2A6 activity in this population. Recent unpublished work has demonstrated that Alaska Natives have a higher frequency of the increase-of-function *CYP2A6* genetic variants rs56113850, rs57837628, rs7260629, rs7259706, rs150298687, and rs8192733 compared to White and African American populations, who have lower CYP2A6 enzyme activity.

Two American Indian populations, the Northern Plains tribal population of South Dakota and the Southwest tribal population of Arizona, have *CYP2A6* allele frequencies that are distinct from White populations, and which also differ significantly between these two tribal groups [42]. Compared to White individuals, both American Indian populations have a higher frequency of the increase-of-function CYP2A6*1B variant and the reduce-of-function CYP2A6*9 variant. The CYP2A6*1B allele is significantly more common in the Northern Plains, whereas the CYP2A6*9 SNP is present at a significantly higher frequency in the Southwest tribal population. Further, the overall frequency of *CYP2A6* genotype reduced metabolizers (i.e., individuals whose *CYP2A6* genotype is composed of one or more decrease or loss-of-function *CYP2A6* genetic variants) is lower in the Northern Plains than the Southwest (28% vs. 42%). Smokers in the Northern Plains tribal population exhibit significantly higher CYP2A6 activity (measured by their NMR), compared to Southwest smokers, and compared to smokers of other ethnic/racial groups (Alaska Natives, White individuals, and African Americans); this difference remains significant even when excluding all individuals who were *CYP2A6* genotype reduced metabolizers (i.e., when comparing CYP2A6*1/*1 genotype smokers only). This suggests that the lower frequency of *CYP2A6* genotype reduced metabolizers in the Northern Plains, compared to the Southwest, does not account for the Northern Plains’ greater overall CYP2A6 activity. It is likely that there are other factors that contribute to the high CYP2A6 activity in the Northern Plains American Indian population, as observed among Alaska Natives [56]. The source of the much faster metabolism among specific ethnic groups necessitates further investigation due to the potential health issues associated with faster metabolism (see below). 

### 2.3. Factors That Regulate CYP2A6 Activity

In addition to the contribution of *CYP2A6* genetics to variation in activity, the expression and activity of CYP2A6 can be induced and inhibited by a variety of substances, including drugs, endogenous substances, and dietary constituents. The primary perpetrators of CYP2A6 induction and inhibition, as well as some possible indirect influences on CYP2A6 activity, are described below.

#### 2.3.1. Inducers

**Drugs**. Phenobarbital, dexamethasone, and rifampin are medications that have been shown to induce CYP2A6 [9,57,58]. The induction of CYP2A6 enzyme activity likely occurs through increased CYP2A6 transcription, mediated by these therapeutics via activation of the following nuclear hormone receptors: constitutive androgen receptor (CAR), pregnane X receptor (PXR), peroxisome proliferator-activated receptor-γ coactivator 1α (PGC-1α), hepatocyte nuclear factor 4 alpha (HNF4α), and the glucocorticoid receptor [59,60,61]. 

**Endogenous Substances**. There are gender differences in CYP2A6 enzyme activity, with females exhibiting faster CYP2A6 activity relative to males (based on measurement of nicotine clearance, cotinine clearance, fractional conversion of nicotine to cotinine, and the NMR) [62]; this likely results from the induction of *CYP2A6* transcription by the endogenous hormone, estrogen. Higashi and colleagues [7] demonstrated that the transcription of the *CYP2A6* gene is induced by estrogen such that it binds to and activates the estrogen receptor, which relocates to the nucleus where it binds to an estrogen response element (ERE), located several kb upstream of the *CYP2A6* gene. The ERE transactivates the *CYP2A6* promoter, inducing the transcription of the gene. As demonstrated in two separate human liver banks, CYP2A6 mRNA and protein levels are higher among female than male liver donors, supporting the notion that gender differences in CYP2A6 occur at the level of gene expression [4,25]. Clinical evidence also supports the involvement of estrogen in the induction of CYP2A6. For example, among women, using estrogen-containing oral contraceptives is associated with greater CYP2A6 activity relative to not using oral contraceptives or using estrogen-free therapies [62]. Use of estrogen-containing birth control pills and hormone replacement therapy are associated with 20% and 30% higher CYP2A6 activity (the NMR), respectively, compared to no drug [63]. Further, there is no difference in the CYP2A6 activity among men or menopausal/post-menopausal women not on estrogen containing hormone replacement [62]. 

**Dietary Substances**. Broccoli consumption, in large quantities (500 g for 6 days), is associated with increased CYP2A6 enzyme activity. The effect of broccoli on CYP2A6 was quantified using the ratio of 1,7-dimethylurate (17U) to 1,7-dimethylxanthine (17X) [6], two metabolites of caffeine, as the conversion of 17X to 17U is mediated, in part, by CYP2A6 (described later in this review) [16]. 

#### 2.3.2. Inhibitors

**Drugs**. Both mechanism-based and competitive inhibitors act on the CYP2A6 enzyme. 8-Methoxypsoralen, also known as methoxsalen, and selegiline are examples of mechanism-based inhibitors (MBIs) of CYP2A6 [8,64]. Methoxsalen is used to treat skin conditions, including psoriasis and eczema [65,66]. Selegiline is a monoamine oxidase (MAO) inhibitor that is used in the early treatment of Parkinson’s disease [67,68]. As MBIs, methoxsalen and selegiline are metabolically activated by CYP2A6, and their products subsequently irreversibly bind to and inhibit CYP2A6 [69]. Another MAO inhibitor, tranylcypromine, acts as a competitive inhibitor of CYP2A6 in vitro with coumarin used as a substrate of CYP2A6 [70]. Competitive inhibition is characterized by reversible binding of the inhibitor to the substrate-binding site of the enzyme, such that inhibition can be overcome using a high concentration of substrate. Another competitive CYP2A6 inhibitor is the anti-fungal ketoconazole, which inhibits CYP2A6-mediated 7-hydroxycoumarin formation in human liver microsomes [71], although the degree of inhibition of CYP2A6 is much lower than that of CYP3A4 by ketoconazole [72].

**Endogenous Substances**. Tryptamine, an indole alkaloid found at low concentrations in the brain, which is a substrate of MAOs [73], competitively inhibits CYP2A6 [70]. At high concentrations, other neurotransmitters, including dopamine and serotonin, also appear to inhibit CYP2A6 activity (coumarin 7-hydroxylation) in vitro [74]. 

**Dietary Substances**. Exposure to several CYP2A6 inhibitors can also occur through diet. Menthol, grapefruit juice, caffeic acid, p-coumaric acid, quercetin, and cinnamaldehyde are substances present in food products that inhibit CYP2A6 enzyme activity. Menthol, which is used to add flavour to certain foods, toothpastes, cigarettes, and other products, has been shown to inhibit in vitro nicotine and coumarin metabolism (a measure of CYP2A6 activity) in human liver microsomes [75]. This is supported by in vivo assessments in which the rate of nicotine metabolism to cotinine was slower among individuals smoking mentholated cigarettes for one week, compared to when these individuals smoked nonmentholated cigarettes for the same duration [76]. Similarly, grapefruit juice consumption, compared to water, is associated with decreased conversion of nicotine to cotinine, lower nonrenal nicotine clearance, and an increase in nicotine and cotinine renal clearance [5], suggestive of reduced/inhibited CYP2A6 enzyme activity. Three compounds that are present in coffee, caffeic acid, p-coumaric acid, and quercetin, have been shown to inhibit CYP2A6 enzyme activity (nicotine metabolism to cotinine) in vitro in human liver microsomes and CYP2A6 supersomes [77]. However, their relatively low inhibitory potency, and the relatively low levels of each compound in coffee, suggest that they are not likely to substantially inhibit CYP2A6 activity in vivo following typical daily levels of coffee consumption. Additionally, cinnamaldehyde, the primary component of cinnamon that gives it flavour and aroma, acts as a mechanism-based inhibitor of CYP2A6 activity in vitro, as measured by the inhibition of coumarin and letrozole metabolism in human liver microsomes and CYP2A6 supersomes [78]. 

### 2.4. Other Influences on CYP2A6

Additional factors may indirectly influence CYP2A6 activity. For example, age is positively, albeit weakly, associated with CYP2A6 protein levels and enzyme activity (nicotine and coumarin metabolism) in a human liver bank [4]. In contrast, a lower rate of nonrenal nicotine clearance has been observed in subjects aged 65 and older, compared to subjects in the 22–43 age group [79], although this may be a product of physiological effects of aging, such as reduced liver blood flow. Other in vitro [25] and in vivo [80] studies do not support a relationship between age and CYP2A6 activity. Additional investigations of the impact of age, and age-related changes, on CYP2A6 enzyme activity may clarify this relationship.

The activity of the CYP2A6 enzyme may also be dependent on the levels and activity of the hepatic cytochrome P450 oxidoreductase (POR) enzyme, which is vital to the catalytic function of all drug-metabolizing CYPs. POR is a membrane-bound enzyme that donates electrons from NADPH to CYPs during their catalytic cycle [81]. The fundamental role of POR in CYP enzyme function is illustrated by the substantial decrease in CYP activity in hepatic POR-deficient mice [82,83]. As POR protein levels are moderately positively correlated with CYP2A6 enzyme activity in vitro (nicotine and coumarin metabolism) [4], variation in the expression or activity of POR may impact CYP2A6 catalytic activity. In fact, variation in the polymorphic gene that encodes the POR enzyme is associated with variation in CYP2A6 enzyme activity such that CYP2A6 normal metabolizers (i.e., individuals who do not possess any decrease- or loss-of-function *CYP2A6* genetic variants) who possess the A503V *POR* variant exhibit faster CYP2A6 activity (measured by the NMR) compared to individuals without this variant [84]. 

Another enzyme that may play a role in variable CYP2A6 enzyme activity is aldo-keto reductase 1D1 (AKR1D1). AKR1D1 is involved in the synthesis of bile acids and the reduction of some steroid hormones, which have been implicated in the transcriptional regulation of CYPs via activation of nuclear hormone receptors, such as PXR and CAR [85,86,87]. Through the generation of a CYP regulatory subnetwork, AKR1D1 was identified as an important regulator of hepatic CYP expression [88]. Follow up in vitro work using human hepatocytes indicated that the overexpression of AKR1D1 was associated with greater expression of multiple CYPs, including CYP2B6, CYP2C8, CYP2C9, CYP2C19, and CYP3A4 [89]. Likewise, the knockdown of AKR1D1 expression resulted in decreased CYP expression levels. Although CYP2A6 was not included in the array of CYPs that were investigated in this study, AKR1D1 and CYP2A6 mRNA, protein, and enzyme activity were moderately positively correlated, and AKR1D1 mRNA expression accounted for a significant proportion (16%) of the variation in CYP2A6 mRNA levels in a human liver bank [4]. These findings suggest that AKR1D1 may indirectly regulate CYP2A6 transcription through its role in bile acid and steroid hormone synthesis and their subsequent activity toward nuclear hormone receptors.

## 3. CYP2A6 and Nicotine Metabolism

### 3.1. Pathways of Nicotine Metabolism

**Figure 1:**
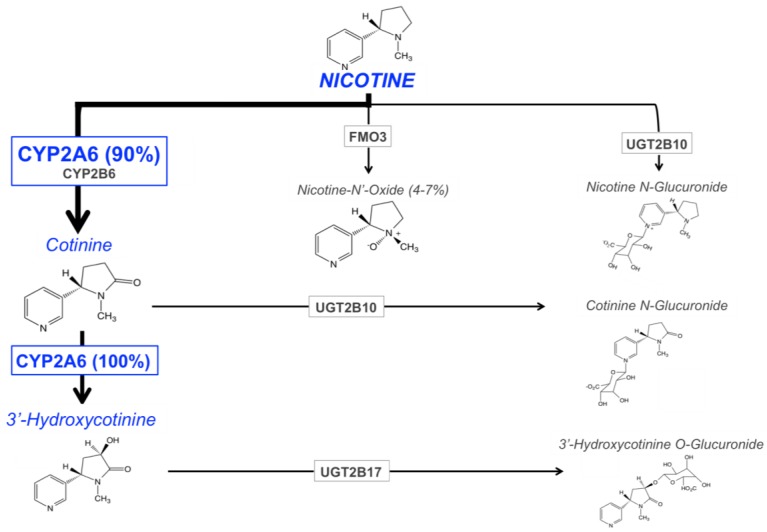
The major pathways of nicotine metabolism and clearance. The bolded arrow represents the predominant pathway of nicotine metabolism (CYP2A6 is responsible for 90% of nicotine’s metabolism to cotinine). Adapted from Hukkanen et al., 2005 [103] and Tanner et al., 2015 [41].

The main enzymes responsible for nicotine metabolism are cytochrome P450 enzymes (CYPs; 70–80% of nicotine metabolism) [90], flavin-containing monooxygenase 3 (FMO3; 4–7%), and uridine diphosphate-glucuronosyltransferases (UGTs; 3–5%) [91,92] (Figure 1). The rate of nicotine inactivation and clearance is primarily dependent on the activity of the CYP2A6 enzyme. Cotinine is the main metabolite of nicotine, with 70 to 80% of nicotine being converted to cotinine; CYP2A6 accounts for approximately 90% of this metabolism [12]. This metabolic conversion occurs in two steps. First, nicotine is metabolized to the nicotine-Δ^1′(5′)^-iminium ion by CYP2A6 (the rate-limiting step), followed by the metabolism of this intermediate metabolite to cotinine by the cytosolic enzyme, aldehyde oxidase [93]. Cotinine is then further metabolized to *trans*-3′hydroxycotinine (3HC) in a process that is entirely mediated by the CYP2A6 enzyme [17]. The half-lives of nicotine, and its two primary metabolites, cotinine and 3HC, are approximately 1 to 2, 16 to 18, and 6 to 7 h, respectively [94,95,96]. The long half-life of cotinine, and the formation dependence of 3HC from cotinine, results in stable relative levels of these metabolites over time in regular smokers. For these reasons, the ratio of these metabolites (3HC/COT, also referred to as the nicotine metabolite ratio, NMR), in the saliva, plasma, or urine of smokers, can be used as a biomarker of CYP2A6 enzymatic activity, and a measure of the rate of nicotine clearance [18]. The NMR is discussed in further detail in the following section. 

The remaining portion of nicotine metabolism to cotinine (approximately 10%) is mediated by CYP2B6 [97,98]. Nicotine is also metabolized to nicotine *N*′-oxide by FMO enzymes, however only 4 to 7% of nicotine dose recovered in the urine is in the form of nicotine *N*’-oxide [91,92]. The final class of nicotine metabolizing enzymes are UGTs, with multiple UGT isoforms contributing to the *N*-glucuronidation of nicotine, which is then excreted in the urine [91,92]. There is evidence for the involvement of UGT1A1, UGT1A4, UGT1A9, UGT2B7, and UGT2B10 in the glucuronidation of nicotine [99,100,101], with UGT2B10 playing the most prominent role due to its high affinity for nicotine (*K_m_* 0.29 mM) and high level of expression in human liver, relative to other UGTs [99,102]. 

### 3.2. Nicotine Metabolite Ratio

As discussed previously, the major nicotine metabolic pathway is the inactivation to cotinine, followed by further metabolism of cotinine to 3HC, processes which are 90% and 100% mediated by the CYP2A6 enzyme, respectively [12,17]. The ratio of these primary metabolites, 3HC/COT (the nicotine metabolite ratio, NMR), is used as a phenotypic biomarker of CYP2A6 enzymatic activity and the rate of nicotine metabolism [18]. The rate of nicotine metabolism, and thus the NMR, varies widely across individuals and is associated with variable smoking behavior (discussed in the next section). The NMR is highly correlated with overall rate of nicotine clearance [18], which results from the primary role CYP2A6 plays in nicotine’s metabolism (70–80%), and the major contribution of metabolism to overall nicotine clearance (95% metabolic vs. 5% renal clearance) [103]. Further evidence for the NMR as a biomarker of CYP2A6 activity is the lack of 3HC made by subjects with the CYP2A6*4/*4 genotype (full gene deletion), indicating that there is no conversion of cotinine to 3HC in these CYP2A6 null individuals [18]. The half-lives of cotinine and 3HC are 16 to 18 and 6 to 7 h, respectively. The formation dependence of 3HC, coupled with the longer relative half-life of cotinine versus 3HC, contributes to the stability of the NMR over time in regular smokers [104,105] irrespective of sampling time of day [106], and across a 44-week repeated sampling period [107]. Measurements of the NMR in different biological matrices (saliva, plasma, blood, and urine) are also consistent (correlations ranging from *r* = 0.76–0.95) [107], as is the measurement of the NMR between laboratories [108], further contributing to the utility of this biomarker. Altogether, these findings indicate that the metabolic clearance rate of nicotine, indicative of CYP2A6 enzyme activity, in a regular smoker can be reliably and reproducibly estimated by measuring the NMR through obtaining a single biological sample. 

In addition to the NMR, other ratios of nicotine and its metabolites can also been used to quantify CYP2A6 enzyme activity. Among non-smokers or abstinent smokers, COT/nicotine can be measured in plasma or urine following timed administration of nicotine. The ratio of total 3HC (i.e., 3HC + 3HC-glucuronide) to COT can be measured in the urine of smokers; this measure is highly correlated with plasma 3HC/COT. 

## 4. CYP2A6 and Smoking, Cessation, and Cancer

### 4.1. Smoking Behavior

Nicotine is the primary psychoactive component of cigarette smoke, responsible for eliciting reward from smoking and for eventual withdrawal during smoking abstinence. The duration of nicotine’s action in the central nervous system (CNS), which is primarily mediated by the rate of metabolism and clearance by the CYP2A6 enzyme, is important in the modulation of smoking behaviors. Several stages of smoking, and the impact of CYP2A6, will be outlined in this section.

**Nicotine Dependence**. Among adult regular smokers, nicotine dependence is associated with *CYP2A6* genotype and the rate of nicotine metabolism, however findings are inconsistent. In several investigations, smokers with *CYP2A6* reduce-of-function genotypes or slower CYP2A6 activity (lower NMR quartiles) exhibited lower FTND (Fagerström Test for Nicotine Dependence) scores, a measure of the degree of nicotine dependence, compared to faster nicotine metabolizers [109,110,111]. Conversely, multiple studies indicated no difference in FTND among different *CYP2A6* genotype or NMR groups [49,112,113]. Because the FTND measure is largely determined by smoking quantity (cigarettes per day, CPD), differences may not be detectable in light smoking populations where smoking intensity plays a larger role than the number of CPD [49]. 

**Smoking Quantity**. Smokers can regulate their tobacco and thus nicotine intake in order to maintain relatively constant circulating nicotine levels throughout the day through altering smoking topography or the number of cigarettes smoked [114]. This occurs to a different extent among smokers, likely due to differences in cigarette craving, which is negatively associated with levels of nicotine in the blood [115]. Accordingly, variation in the rate of nicotine metabolism (CYP2A6 activity) and clearance, primarily mediated by *CYP2A6* genetic variation, is associated with differences in smoking quantity.

CYP2A6 variation is associated with differences in both the number of cigarettes smoked per day and smoking topography (puff volume, duration, and velocity). Multiple studies have shown that smokers with reduce-of-function *CYP2A6* genotypes smoke fewer CPD than those with wild-type genotypes [21,110,116,117]. Smokers with the full *CYP2A6* gene deletion (CYP2A6*4/*4 genotype) smoke significantly fewer CPD compared to smokers with one or two active functional copies of the gene [116]. This relationship also extends beyond the gene deletion. In a meta-analysis of eighteen studies, from multiple ethnic/racial populations, that each assessed the association between *CYP2A6* genotype and CPD, reduced metabolizers (individuals with one or more of the *CYP2A6* alleles CYP2A6*2, CYP2A6*4, CYP2A6*7, CYP2A6*9, CYP2A6*10, CYP2A6*12, CYP2A6*17, CYP2A6*20, CYP2A6*23, CYP2A6*24, CYP2A6*25, CYP2A6*26, CYP2A6*27, CYP2A6*28, CYP2A6*35) smoked significantly fewer CPD compared to normal nicotine metabolizers (individuals with the CYP2A6*1/*1 genotype) [118]. Similar relationships have been demonstrated for NMR (CYP2A6 activity) and CPD [119].

The relationship between smoking quantity and CYP2A6 is also seen when biochemical measures of smoking quantity (plasma cotinine, breath CO) or intensity (CO/cigarette) are used. For example, smokers possessing the *CYP2A6* gene duplication (i.e., possessing more than two copies of the *CYP2A6* gene) have been shown to smoke cigarettes more intensely than smokers with just two functional copies of the gene (CYP2A6*1/*1), as evidenced by a CO/cigarette ratio double that of wild-type smokers [21]. Similarly, when assessing the association between NMR quartiles and smoking quantity, there was a significant stepwise increase in total puff volume and NNAL (a tobacco-specific carcinogen) exposure with each NMR quartile [120]. A similar association was observed between *CYP2A6* genotype group and smoking intensity, measured as mean and total puff volume [121]. 

In lighter smokers, those who consume on average fewer than 10 CPD, these biochemical measures of smoking quantity are superior to CPD due to their higher stringency in differentiating subtle differences in smoking. For example, in a study of adult African American light smokers (≤10 CPD), self-reported CPD did not differ across *CYP2A6* genotype groups or NMR quartiles [122]. Similarly, among Alaska Native light smokers (<10 CPD), there were no significant differences in CPD or chews per day (smokeless tobacco) based on CYP2A6 activity (slower vs. faster NMR group) [123]. However, urinary total nicotine equivalent (TNE) levels were significantly lower for both smokers and smokeless tobacco users in the lower NMR (less CYP2A6 activity) group, indicating that slower nicotine metabolizers had lower levels of tobacco consumption than faster nicotine metabolizers. The TNE biomarker, calculated as the sum of nicotine and all of its metabolites excreted in urine, is a measure of total nicotine intake that is not directly impacted by variation in the rate of nicotine metabolism, as the primary metabolism pathways are accounted for; 88% and 98% of systemic nicotine doses from smoking and nicotine patch, respectively, are accounted for when measuring TNE [91]. This underlies the utility of TNE when comparing individuals with different CYP2A6 enzyme activities, such as those with variable *CYP2A6* genotypes, or when comparing males and females. Together these findings suggest that, when using more sensitive smoking biomarkers, such as TNE, light smokers appear to regulate their smoking quantity and nicotine intake by adjusting their smoking topography (e.g., intensity), as opposed to CPD.

### 4.2. Smoking Cessation

Smokers with different *CYP2A6* genotypes and rates of nicotine metabolism vary in their ability to quit smoking, such that slow nicotine metabolizers are more likely to quit smoking compared to normal metabolizers. Without the use of pharmacological smoking cessation aids, *CYP2A6* genotype slow metabolizers have a greater likelihood of spontaneous quitting compared to smokers with normal *CYP2A6* genotypes (i.e., wild-type, no identified variant alleles) [124,125]. The improved cessation success among slower nicotine metabolizers has been further investigated with relation to cessation pharmacotherapies. A widely used smoking cessation treatment is nicotine replacement therapy (NRT), which comes in the form of nicotine patch, gum, lozenges, nasal spray, and inhaler. Compared to faster nicotine metabolizers (higher NMR, greater CYP2A6 activity), slower metabolizers exhibit higher quit rates following 8 weeks of treatment with nicotine patch [113,126]. Another smoking cessation pharmacotherapy is bupropion, which inhibits dopamine and norepinephrine reuptake and functions as a weak nicotinic acetylcholine receptor (nAChR) antagonist [127]. Bupropion is not metabolized by CYP2A6, but rather by CYP2B6. In contrast to the NRT clinical trials, a study by Chen and colleagues [128] showed, as expected, that there was no association between *CYP2A6* genotype and quitting using bupropion therapy, with a similar decrease in smoking relapse observed among *CYP2A6* genotype slow and fast nicotine metabolizers. In a separate smoking cessation clinical trial, end-of-treatment quit rates decreased with increasing NMR quartiles in the placebo group, whereas quit rates again did not differ according to CYP2A6 activity (NMR quartiles) for subjects taking bupropion. Due to the low quit rates on placebo, bupropion was associated with significantly improved quit rates compared to placebo (34% vs. 10%) for the highest NMR quartile (i.e., fastest nicotine metabolizers, greatest CYP2A6 activity, worst cessation on placebo, similar cessation on bupropion: bupropion significantly superior to placebo for this group) [129]. No significant differences in quit rates from bupropion versus placebo were observed in the three lower NMR quartiles. 

The abovementioned analyses of CYP2A6 and cessation were retrospective analyses of existing clinical trials, whereas a recently completed phase 3 clinical trial demonstrated the utility of the NMR as a predictive biomarker of smoking cessation outcome [130]. In the Pharmacogenetics of Nicotine Addiction Treatment [130] clinical trial (NCT0131001), participants were randomized to treatment groups (placebo, nicotine patch, or varenicline) using prospective stratification based on their pretreatment NMR (representative of their CYP2A6 enzyme activity). The primary hypothesis was the faster metabolizers would have better quit rates on the non-substrate varenicline than on nicotine patch, in contrast to slower metabolizers who would have similar quit rates in both treatment arms. Varenicline is a partial agonist, and competes with nicotine binding, at α4β2 nAChRs, reducing nicotine-evoked dopamine release, the primary reward mechanism of smoking [131]. At end-of-treatment (11 weeks) and six-month follow-up, slower nicotine metabolizers in the PNAT trial exhibited no difference in quit rates between nicotine patch or varenicline, whereas faster metabolizers exhibited significantly higher quit rates on varenicline compared to nicotine patch [130]. Further, among slow metabolizers, number needed to treat (NNT) was similar for nicotine patch and varenicline treatments (10.3 and 8.1, respectively), while the NNT was 26.0 compared to 4.9 for nicotine patch and varenicline, respectively, among normal metabolizers. Varenicline (versus placebo) associated side effects were also more debilitating for slow versus faster metabolizers. Collectively, these data suggest that slower nicotine metabolizers, those with less CYP2A6 enzyme activity, may benefit most from NRT, because of a safer side effect profile and lower cost, whereas faster metabolizers, those with greater CYP2A6 activity, may benefit from bupropion or varenicline. 

### 4.3. Lung Cancer Risk

There are many tobacco-related diseases, which have been associated with variation in CYP2A6, including lung cancer, chronic obstructive pulmonary disease, diabetes, abdominal obesity, and others. However, by far the greatest level of evidence has thus far been with tobacco-related lung cancer risk. This may be in part due to the reliance on GWAS data for associations with many disorders, whereas *CYP2A6* has many copy number variants and low frequency alleles, weakening the utility of GWAS analyses of this gene. Additionally, CYP2A6 plays a dual role in lung cancer risk among smokers via both indirect and direct mechanisms. Smoking fewer CPD is associated with lower lung cancer risk [132], resulting from lower exposure to harmful carcinogens, such as the tobacco-specific nitrosamines *N*-nitrosonornicotine (NNN) and 4-(methylnitrosamino)-1-(3-pyridyl)-1-butanone (NNK) [133]. As slower nicotine metabolism, due to less CYP2A6 enzyme activity, is associated with smoking fewer CPD [118], and lower smoking intensity [120], CYP2A6 activity variation may indirectly influence lung cancer risk through lowering smoking quantity and procarcinogen exposure. However, the CYP2A6 enzyme can act directly via metabolically activating procarcinogenic tobacco-specific nitrosamines NNN and NNK via α-hydroxylation [10,11] suggesting that slower activation may also lower lung cancer risk. Therefore, smokers with slower CYP2A6 activity will have less activation of tobacco-specific nitrosamines, decreasing exposure to these activated lung carcinogens. This notion is supported by the measurement of higher levels of NNN, the parent pro-carcinogen, suggesting lower levels of NNN bioactivation, among smokers who were *CYP2A6* genotype and phenotype reduced compared to normal metabolizers, even when controlling for smoking quantity (urinary TNE) [123]. 

Further support for a direct effect can be found from studies, where after controlling for cigarette consumption (cigarettes per day and/or cigarette pack-years), *CYP2A6* genotype reduced metabolizers, relative to normal metabolizers, continue to exhibit a decreased risk of having lung cancer [110,134]. Additionally, *CYP2A6* genetic variants are associated with lung cancer in a recent GWAS (Transdisciplinary Research in Cancer of the Lung (TRICL) consortium), even when controlling for smoking duration and quantity [44]. The top genome-wide significant SNPs were located within or near the *CYP2A6* gene and remained genome-wide significant after conditioning on known functional *CYP2A6* genetic variants. Several of the top SNPs remained significant when adjusting for smoking status and cigarette pack-years. Furthermore, the NMR (measured as the ratio of urinary total 3HC/COT), which captures more of the functional variation in CYP2A6 activity than currently identified genetic variants alone, as well as potential environmental variation, has also been associated with lung cancer risk in a prospective multi-ethnic cohort study [135]. Even after controlling for level of tobacco consumption, lung cancer risk was greater among smokers with higher NMRs (hazard ratio per unit increase in log-NMR is 1.46, *p* = 0.02). Together these findings highlight the possibility for a more direct role of CYP2A6 in lung cancer risk. Thus, being a slower CYP2A6 metabolizer can reduce both tobacco consumption and procarcinogen activation, resulting in lower lung cancer risk.

## 5. CYP2A6 and Other Clinical Therapeutics

In addition to playing a primary role in the metabolism of nicotine, in turn influencing many smoking behaviors, including quitting smoking, as well as tobacco-related diseases such as lung cancer risk, the CYP2A6 enzyme also metabolizes several other clinically relevant substrates. These include tegafur, letrozole, efavirenz, valproic acid, pilocarpine, artemisinin, and SM-12502. Variation in CYP2A6 enzyme activity, influenced by either *CYP2A6* genetic variation or non-genetic factors, may impact the therapeutic response to these CYP2A6 substrates, but for some genetic variants, such as CYP2A6*17, the functional impact may vary by substrate. Likewise for a few of these substrates, it is not clear whether the impact of the genetic variation in *CYP2A6* is sufficient to be clinically meaningful. Compared to the body of literature describing CYP2A6, nicotine metabolism, and clinically relevant outcomes, evidence is limited for the clinical impact of CYP2A6 variation on the metabolism of these substrates. A summary of the available literature is provided in this section.

### 5.1. Tegafur

Tegafur is a prodrug, used in the treatment of a variety of cancers, that is metabolized primarily by CYP2A6 to the active metabolite 5-fluorouracil [13,136]. As tegafur and other anticancer drugs have narrow therapeutic windows, assessing variation in tegafur metabolism, potentially resulting from variation in CYP2A6 enzyme activity, is important to ensure that an adequate amount of the active 5-fluoruracil is produced to ensure treatment efficacy and to avoid toxicity. 

In a study of 45 human livers, the in vitro formation of 5-fluorouracil from tegafur in human liver microsomes was reduced in donors possessing the CYP2A6*4 gene deletion allele, which also corresponded to significantly lower CYP2A6 mRNA levels in these livers, compared to donors without this variant [137]. Similar findings were demonstrated in vivo in Japanese cancer patients such that individuals with the CYP2A6*4 allele had a higher plasma area under the concentration-time curve from 0 to 10 h (AUC_0–10_) for tegafur and a lower AUC_0–10_ for 5-fluorouracil compared to patients without the gene deletion; this suggests that CYP2A6*4 individuals are experiencing less exposure to the active metabolite 5-fluorouracil, potentially resulting in reduced therapeutic efficacy [138]. Similarly, studies of Japanese cancer patients showed that individuals with *CYP2A6* genotypes consisting of two reduce-of-function variants exhibited lower tegafur clearance relative to individuals with one or zero reduce-of-function variants; however, this did not correspond to a lower exposure of patients to 5-fluorouracil, as the overall 5-fluorouracil area under the concentration-time curve did not correlate with that of tegafur [139,140]. Taken together, these findings suggest that a substantial reduction in *CYP2A6* expression and thus enzyme activity, as seen with the CYP2A6*4 gene deletion allele, may be necessary in order to elicit a change in patient exposure to the active metabolite 5-fluorouracil. 

Differences in tegafur metabolism and 5-fluorouracil formation, resulting from variable CYP2A6 activity, can influence treatment outcomes for cancer patients. For example, metastatic gastric cancer patients receiving combination docetaxel and S-1 (a combination of tegafur, 5-chloro-2, 4-dihydroxypyridine, and potassium oxonate) exhibited better treatment response rates and progression-free survival if they did not possess any *CYP2A6* reduce-of-function genetic variants, compared to patients with one or more of these variants [141]. Similarly, following surgery, the relapse-free survival of gastric cancer patients receiving S-1 treatment was higher in those without *CYP2A6* variant genotypes, compared to patients with *CYP2A6* genetic variants [142]. This has also been demonstrated in patients with metastatic colorectal cancer who were treated with combination irinotecan, oxaliplatin, and S-1; patients had greater response to treatment if they did not have any tested *CYP2A6* variant alleles (i.e., faster converters of tegafur to 5-fluorouracil), compared to patients possessing these variants [143]. However, in patients with metastatic biliary tract cancer who were given a combination therapy of oxalipatin and S-1, *CYP2A6* genotype was not associated with clinical efficacy or toxicity, despite *CYP2A6* genotype reduced metabolizers exhibiting higher tegafur C_max_ (maximal plasma drug concentration) and AUC_0–24_, and lower 5-fluoruracil C_max_ and AUC_0–24_, compared to patients without *CYP2A6* reduce-of-function variants [144]. Similar negative results have been observed for other tegafur or S-1 combination therapies [145,146]. These results suggest that the impact of *CYP2A6* genotype on the efficacy of tegafur-containing cancer treatments may be dependent on cancer type as well as the other chemotherapies given in combination with tegafur or S-1. 

### 5.2. Letrozole

Another anticancer drug, letrozole, is metabolized and inactivated by CYP2A6 and CYP3A4 [14]. Letrozole is used in the treatment of estrogen receptor- and progesterone receptor-positive breast cancer in postmenopausal women [147]. *CYP2A6* genotypes with the reduce-of-function variants *4, *7, *9, and *10 were associated with less letrozole and coumarin (CYP2A6-specific substrate) metabolism in human liver microsomes, compared to CYP2A6*1/*1 genotype livers [14]. In a clinical trial comparing letrozole to exemestane treatment in women with breast cancer, plasma letrozole concentrations were significantly associated with *CYP2A6* genotype, such that patients with reduce-of-function genetic variants exhibited higher letrozole concentrations compared to wild-type (CYP2A6*1/*1) patients [148]. Age and body mass index (BMI) were positively and negatively correlated with plasma letrozole concentrations, respectively [148], both of which are factors that have previously been associated with CYP2A6 activity [4,63]. The relationship between *CYP2A6* genotype and letrozole was further demonstrated in a group of healthy postmenopausal women, with women who possess the reduce-of-function *CYP2A6* genetic variants exhibiting decreased letrozole clearance relative to women without these variants [149]. It remains to be seen if this impact of *CYP2A6* genetic variation on letrozole metabolism and clearance significantly impacts treatment efficacy and response in breast cancer patients. 

### 5.3. Efavirenz

Efavirenz is an antiretroviral drug used to prevent and treat HIV/AIDs (human immunodeficiency virus/acquired immunodeficiency syndrome). Plasma efavirenz levels vary widely across patients, with low levels being associated with treatment failure, and high levels associating with side effects such as CNS toxicity [150]. Efavirenz is primarily metabolized to 8-hydroxyefavirenz (77.5% of efavirenz metabolism in vitro) by CYP2B6, and is also metabolized to 7-hydroxyefavirenz (22.5% of efavirenz metabolism in vitro) by CYP2A6 and CYP2B6 [151]. Possibly resulting from the minor role of CYP2A6 relative to CYP2B6 in efavirenz metabolism, and due to small samples sizes or low allele frequencies, *CYP2A6* genetic variants are not significantly associated with efavirenz plasma levels in several studies [152,153,154,155,156]. However, when subjects are stratified based on *CYP2B6* genotype, *CYP2A6* genotype is associated with plasma efavirenz levels; within the reduced CYP2B6 activity group (according to genotype), the CYP2A6*9 allele is associated with increased efavirenz levels [157], and *CYP2A6* reduce-of-function genotypes are associated with lower levels of 7-hydroxyefavirenz [15]. CYP2A6 activity appears to become more important to efavirenz metabolism when the contribution of the major metabolizing enzyme, CYP2B6, is reduced. 

There are also several clinical studies that have shown a significant association of *CYP2A6* genotype with efavirenz levels during treatment, when not stratifying by *CYP2B6* genotype group. For example, Kwara et al., 2009 [158] demonstrated that *CYP2A6* genotype reduced metabolizers (those with CYP2A6*9 and/or CYP2A6*17 alleles) have significantly higher plasma efavirenz levels compared to individuals without the CYP2A6*9 and CYP2A6*17 genetic variants. The same was true for patients possessing the CYP2B6*9/*9 genotype and those with one or two copies of the UGT2B7*1a allele, while the UGT2B7*2 allele was associated with decreased efavirenz levels [158]. Similarly, CYP2A6*9, CYP2B6*9, and CYP2B6*18 were each associated with lower plasma efavirenz levels in a sample of more than 500 HIV-infected patients [159]. The resulting increase in patients’ exposure to efavirenz may negatively influence treatment adherence. *CYP2A6* reduce-of-function genotypes were associated with greater likelihood of discontinuing efavirenz treatment in HIV-infected patients, potentially resulting from higher efavirenz plasma levels leading to an increase in adverse side effects, such as CNS and neuropsychiatric reactions [150,160,161]. Overall, a combination of genetic variation in *CYP2A6*, *CYP2B6*, as well as other drug metabolizing enzymes that play minor roles in efavirenz metabolism, may impact the efficacy or toxicity of efavirenz treatment for HIV through altering patients’ exposure to the drug. 

### 5.4. Valproic Acid

The CYP2A6 enzyme is involved in the metabolism of valproic acid, an antiepileptic drug, to 4-ene-valproic acid, 3-hydroxy-valproic acid, 4-hydroxy-valproic acid, and 5-hydroxy-valproic acid [162]. Valproic acid treatment has been associated with hepatotoxicity [163], potentially mediated by its reactive metabolite 4-ene-valproic acid [164]. Although CYP2A6 contributes to 4-ene-valproic acid formation from valproic acid, the major metabolite of CYP2A6-mediated valproic acid metabolism is 3-hydroxy-valproic acid, with CYP2C9 functioning as the major enzyme involved in the formation of 4-ene-valproic acid from the parent compound [162,165].

Variation in CYP2A6 expression or activity may be associated with altered valproic acid exposure. For example, among patients treated with valproic acid for epilepsy, CYP2A6*1/*4 and CYP2A6*4/*4 genotypes were associated with higher plasma valproic acid concentrations compared to non-CYP2A6*4 genotypes [166]. A similar association was observed for CYP2B6*6 and CYP2C9*3 and higher valproic acid concentrations [166]. However, the CYP2A6*4 deletion variant was not associated with any change in 4-ene-valproic acid formation or liver dysfunction in 102 patients taking valproic acid for epilepsy [167]. 

### 5.5. Pilocarpine

Pilocarpine, used in the treatment of glaucoma and dry mouth (xerostomia), is primarily metabolized by CYP2A6 to 3-hydroxypilocarpine [168]. In vitro studies have shown that pilocarpine has a high affinity for CYP2A6, and competitively inhibits coumarin 7-hydroxylation in human liver microsomes [169,170]. *CYP2A6* reduce-of-function genotypes were associated with decreased pilocarpine metabolism to 3′-hydroxypilocarpine in healthy Japanese males given oral pilocarpine hydrochloride; subjects possessing two decrease- or loss-of-function *CYP2A6* alleles (combinations of CYP2A6*4, CYP2A6*7, CYP2A6*9, and CYP2A6*10), comprising the “poor metabolizer” group, had higher C_max_ and AUC and lower apparent clearance (CL/F) for pilocarpine, and lower C_max_ and AUC for 3-hydroxypilocarpine, compared to the “non-poor metabolizer” group [171]. As this study was conducted in healthy volunteers, it remains to be seen if *CYP2A6* genetic variation and the resulting change in pilocarpine levels are associated altered treatment efficacy or related side effects. 

### 5.6. Artemisinin and Artesunate

The antimalarial drugs, artemisinin and artesunate, are metabolized by CYP2A6. Artemisinin is metabolically inactivated in vitro by CYP2B6 and CYP3A4, with a minor contribution of CYP2A6 [172], whereas, CYP2A6 is the primary enzyme involved in the metabolic activation of the artemisinin derivative, prodrug artesunate, to dihydroartemisinin [173]. Genetic variation in the *CYP2A6* gene that decreases CYP2A6 activity may result in less artemisinin inactivation or decreased formation of the pharmacologically active dihydroartemisinin metabolite from artesunate, potentially resulting in decreased therapeutic efficacy. Conversely, increase-of-function *CYP2A6* genetic variants, such as the CYP2A6*1B allele, may be associated with increased dihydroartemisinin formation and adverse effects. For example, in 24 healthy Malaysian subjects, the incidence of adverse drug reactions in response to artesunate and amodiaquine treatment was higher among subjects with the CYP2A6*1B genetic variant, compared to those without this allele [174]. In a study of 71 Burmese subjects with acute uncomplicated *Plasmodium falciparum* malaria, the individual with the CYP2A6*1/*4 genotype exhibited higher artesunate (parent) and lower dihydroatremisinin (metabolite) concentrations relative to individuals with the CYP2A6*1A/*1A, CYP2A6*1A/*1B, and CYP2A6*1B/*1B genotypes [175]. This study did not assess several other *CYP2A6* reduce-of-function alleles, including CYP2A6*7, CYP2A6*8, CYP2A6*9, CYP2A6*10, and CYP2A6*12, and the assessment of these additional genetic variants in future studies may increase the power to compare artesunate and dihydroartemisinin concentrations and treatment response across *CYP2A6* genotype groups. 

### 5.7. SM-12502

SM-12502, also known as (+)-cis-3,5-dimethyl-2-(3-pyridyl)thiazolidin-4-one hydrochloride, is a platelet activating factor receptor antagonist [176]. In vitro assessments have indicated that SM-12502 is metabolized primarily by CYP2A6 to S-oxide; S-oxide formation from SM-12502 was significantly inhibited by coumarin, a CYP2A6-specific substrate, and there was strong correlation between coumarin 7-hydroxylation and SM-12502 S-oxidation [177]. In Japanese subjects, slower metabolism of SM-12502 was associated with *CYP2A6* gene deletions present in three subjects [178]. Additional studies assessing more *CYP2A6* genetic variants are needed to evaluate the impact on SM-12502 plasma concentrations and treatment efficacy. 

## 6. CYP2A6 and Dietary Substrates

### 6.1. Caffeine

Although CYP1A2 is the primary enzyme involved in caffeine metabolism [16], CYP2A6 also plays a role in this metabolism pathway; caffeine is metabolized to 1,7-dimethylxanthine (17X) by CYP1A2, and 17X is converted to 1,7-dimethylurate (17U) by both CYP1A2 and CYP2A6 [179]. Further supporting the role of CYP2A6 in the metabolism of 17X to 17U, *CYP2A6* reduce of function genetic variants have been associated with less metabolism of 17X to 17U in vitro in human liver microsomes and in vivo in subjects given oral caffeine as a probe or consuming dietary caffeine [179,180,181,182,183]. For example, when caffeine was given as a probe, individuals possessing the CYP2A6*2, CYP2A6*4, and CYP2A6*9 reduce-of-function genetic variants exhibited significantly lower urinary 17U/17X ratios compared to subjects without these variants [182]. Similar to the NMR, the ratio of caffeine’s metabolites 17U/17X may be a useful CYP2A6 activity phenotype [180,181,182,183,184,185].

### 6.2. Tyrosol

The dietary phenol tyrosol, found in red wine, olives, and olive oil, is metabolized to hydroxytyrosol by CYP2A6, with minor contributions of CYP2D6 and CYP3A4 [186]. Both tyrosol and hydroxytyrosol have antioxidant effects; however, the metabolite, hydroxytyrosol, appears to exhibit greater antioxidant activity than tyrosol [187], suggesting that individuals with faster CYP2A6 enzyme activity and associated genotypes may benefit most from consumption of tyrosol-containing foods.

## 7. Conclusions and Areas of Future Research

CYP2A6, primarily known for its role in nicotine metabolism and smoking phenotypes, is also a key enzyme in the metabolism of other clinically important drugs and procarcinogens, and plays a role in the metabolism of some dietary substrates. Variation in CYP2A6 enzyme activity primarily results from variation in the highly polymorphic *CYP2A6* gene, but can also occur in response to induction or inhibition by drugs, endogenous compounds, and dietary substances. Assessment of these factors, particularly *CYP2A6* genetic variation, will likely be important in the optimization of smoking cessation treatment, as well as in the optimization of therapeutic efficacies and avoidance of adverse drug reactions for other clinically relevant substrates. Therefore, future research efforts should focus on fully characterizing the functional genetic variation at the *CYP2A6* gene locus, and identifying the impact of this functional variation on smoking and treatment outcomes. Recent modified approaches to next-generation sequencing of the *CYP2A6* gene have proven useful in identifying and characterizing novel genetic variants, and these technologies continue to improve and decrease in cost. As we advance our knowledge of variation in CYP2A6 activity, we should extend these findings across ethnically diverse populations, as *CYP2A6* genetic variation and the rate of nicotine metabolism vary widely across these groups.

## Figures and Tables

**Table 1:** Summary of *CYP2A6* genetic variants (minor allele frequency (MAF) > 1%, functionally significant variants only) and their impact on CYP2A6 expression and activity (nicotine metabolism) ^b^.

CYP2A6 Genetic Variant	rs ID	CYP2A6 Region	Genetic Impact	Functional Impact on CYP2A6 ^a^	Allele Frequency (%)					
					White	African	Asian	Alaska Native	American Indian	
									Northern Plains	Southwest
*1B	N/A	3′-UTR	58 bp gene conversion with *CYP2A7*	Increased mRNA stability	28–35	11–18	26–57	65	69.7	61.6
*1X2A and *B*	N/A	intron 8 and 5.2–5.6 kb 3′	*CYP2A6* gene duplications	Increased mRNA expression	0–1.7	0	0–0.4	0	–	–
*2	rs1801272	Exon 3	Nonsynonymous, L160H	Substantially decreased enzyme activity	1.1–5.3	0–1.1	0	0.4	0.3	0.6
*4	N/A	N/A	*CYP2A6* gene deletion	No mRNA expression	0.1–4.2	0.5–2.7	4.9–24	15	1.6	0.3
*5	rs5031016	Exon 9	Nonsynonymous, G479V	Decreased enzyme activity	0–0.3	0	0–1.2	–	–	–
*7	rs5031017	Exon 9	Nonsynonymous, I471T	Decreased enzyme activity	0–0.3	0	2.2–13	0	0	0
*9	rs28399433	5′	Promoter SNP, interrupts TATA box (A>C)	Decreased mRNA expression	5.2–8.0	5.7–9.6	16–22	8.9	11.9	20.9
*10	rs5031017, rs28399468	Exon 9	Nonsynonymous, I471T, R485L	Inactive enzyme	0	0	0.4–4.3	1.9	–	–
*12	esv2663194	N/A	Exons 1–2 from *CYP2A7*, exons 3–9 from *CYP2A6*, 10 amino acid substitution	Decreased enzyme activity	0–0.3	0–0.4	0–0.8	0.4	0.3	0.3
*17	rs28399454	Exon 7	Nonsynonymous, V365M	Substantially decreased enzyme activity	0	7.1–11	0	0	0	0
*18	rs1809810	Exon 8	Nonsynonymous, Y392F	Decreased enzyme activity	1.1–2.1	0	0–0.5	–	–	–
*20	N/A	Exon 4	Two-nucleotide deletion, frame shift, truncated protein	Substantially decreased protein levels	0	1.1–1.7	0	–	–	–
*21	rs6413474	Exon 9	Nonsynonymous, K476R	Decreased enzyme activity	0–2.3	0–0.6	0–3.4	–	–	–
*23	rs56256500	Exon 4	Nonsynonymous, R203C	Decreased enzyme activity	0	1.1–2.0	0	–	–	–
*24	rs72549435, rs143731390	Exon 2, 9	Nonsynonymous, V110L, N438Y	Decreased enzyme activity	0	0.7–2.3	0	–	–	–
*25	rs28399440	Exon 3	Nonsynonymous, F118L	Decreased enzyme activity	0	0.5–1.2	0	–	–	–
*28	rs28399463	Exon 8	Nonsynonymous, N418D, E419D	Decreased enzyme activity	–	0.9–2.4	–	–	–	–
*35	rs143732390	Exon 9	Nonsynonymous, N438Y	Decreased enzyme activity	0	2.5–2.9	0.5–0.8	0	0	0.3
**N/A**	rs56113850	Intron 4	Non-coding SNP (T>C)	Increased protein expression and enzyme activity; top hit in multiple GWASs of NMR	56–59	39	29	72	68	65
**N/A**	rs113288603	5′	Non-coding SNP (C>T)	Decreased enzyme activity	9–15	12	23	–	–	–
**N/A**	rs12459249	3′	Non-coding SNP (T>C)	Increased enzyme activity	68	69–66	41	–	–	–
**N/A**	rs111645190	5′	Non-coding SNP (G>A)	Decreased enzyme activity; may tag the *17 variant	0	14	0	–	–	–
**N/A**	rs57837628	5′	Non-coding SNP (A>G)	Increased protein expression and enzyme activity	49–54	17	29	70	73	58
**N/A**	rs7260629	5′	Non-coding SNP (T>G)	Increased protein expression and enzyme activity	69–72	71	74	83	–	–
**N/A**	rs7259706	5′	Non-coding SNP (C>T)	Increased protein expression and enzyme activity	69–70	73	73	83	–	–
**N/A**	rs150298687	5′	Non-coding SNP (T>C)	Increased protein expression and enzyme activity	58–63	46	45	81	–	–
**N/A**	rs28399453	Intron 6	Non-coding SNP (G>A)	Increased protein expression and enzyme activity	6–7	0	0	3	0	0.3
**N/A**	rs8192733	3′-UTR	Non-coding SNP (G>C)	Increased protein expression and enzyme activity	47–48	23	51	66	–	–

^a^ Functional impact toward nicotine metabolism (nicotine C-oxidation); ^b^ This table has been adapted from Tanner et al., 2015 [41], with additional data from Loukola et al., 2015 [3], Tanner et al., 2017 [42], the 1000 Genomes dataset [43], and unpublished work. Additional references for specific variants are provided in the text. rsID: reference SNP cluster ID, N/A: not applicable, SNP: single nucleotide polymorphism, GWASs: genome-wide association studies.
